# Communicating With Children With Cancer: Development of a Chatbot-Based Educational Support Tool

**DOI:** 10.1007/s13187-025-02711-1

**Published:** 2025-10-11

**Authors:** Eliana Silva, Paulo Pinto, Luís Paulo Reis

**Affiliations:** LIACC/FEUP, Artificial Intelligence and Computer Science Laboratory, Faculty of Engineering, University of Porto, Porto, 4200-465 Portugal

**Keywords:** Cancer, Chatbot, Artificial intelligence, Communication

## Abstract

Effective communication between parents and children is crucial in reducing distress when a child has cancer. Technology, particularly chatbots, can facilitate this communication and provide parents with support. We have developed a chatbot to support parents of children with cancer. Using advanced natural language processing, it provides empathetic, age-appropriate, and informative responses. We created the chatbot using the Dialogflow platform and integrated it into a mobile app and website to ensure broad accessibility. This multi-platform integration provides consistent support, regardless of the device or location used. We conducted a qualitative comparative analysis of our chatbot against other chatbots, such as ChatGPT-4 and Microsoft Copilot. This analysis was based on five standard criteria for the human evaluation of chatbots. In general, our chatbot offered more personalized and targeted support for childhood cancer. Unlike other systems that provide generic responses, our chatbot uses a friendlier tone and tailors its responses based on factors such as the child’s age. This project represents a significant advancement in the use of technology to address the complex communication needs associated with childhood cancer. To our knowledge, it is the first chatbot designed specifically for Portuguese-speaking parents. It offers parents personalized, accessible support and provides a scalable, cost-effective solution that complements the efforts of healthcare professionals. In settings with limited resources, the chatbot is an invaluable tool for bridging the gap between medical consultations.

## Introduction

Childhood cancer is a global health issue and a leading cause of mortality in children (any person under the age of 18 [1]). Treatment poses significant medical and emotional challenges for patients and their families [2, 3]. Despite improved survival rates, cancer treatment remains difficult, impacting the quality of life due to symptoms like pain, fatigue, and anxiety [4]. Effective communication is critical to the treatment outcomes and emotional well-being of child patients and their families [2, 3]. Children, often excluded from discussions, want honest information but prefer it filtered through their parents. In general, parents prefer to get information from a trusted health professional rather than searching the Internet. Internet research was seen as unreliable and daunting, with parents expressing fear of what they might discover, uncertainty about the accuracy of online information, and being overwhelmed by the amount of information available [5]. However, parents of pediatric oncology patients conducted health-related research at a rate more than twice that of the general population (13% versus 5% of all Google searches). Searches increased about a month after a cancer diagnosis [6]. Over time, emotional strain may hinder parent–child communication, making tailored interventions necessary. This underscores the need for tailored interventions to enhance parent–child communication [7, 8]. According to a recent systematic review of the information needs of relatives of childhood cancer patients and survivors, families seek information in several key areas, including medical details, the effects of cancer and its treatments, lifestyle adjustments, family dynamics, and support resources. The authors emphasized the need to develop personalized information resources that assess the specific needs of relatives at diagnosis, continually reassess these needs over time, and provide relevant information accordingly [9].

Chatbots, which simulate human conversation through natural language processing, emerge as valuable tools in healthcare [10]. They offer benefits such as 24/7 availability, improved engagement, and support for tasks like diagnosis, treatment, and education [11]. In childhood cancer, chatbots can enhance communication, provide personalized recommendations, and support symptom tracking. However, concerns about security, accuracy, and the potential to replace human interactions remain [12]. Regulatory frameworks, such as the EU’s Artificial Intelligence Act, aim to address these challenges by ensuring transparency, data protection, and oversight for AI applications in healthcare [13]. Existing chatbots like ChatGPT and Copilot face limitations in healthcare contexts. ChatGPT may provide incorrect information, experience hallucinations, and lose the thread of long conversations. This can be problematic in sensitive settings such as childhood cancer [14]. Copilot, while useful for coding, lacks the ability to understand user intentions and struggles with natural language processing, making it less effective in healthcare [15]. Both systems highlight the need for more refined, context-aware solutions in healthcare communication, particularly in emotionally sensitive areas like childhood cancer. We aim to present a chatbot developed by a multi-disciplinary research team consisting of computer engineers and psychologists. Designed to support parents in effectively communicating complex medical information to children diagnosed with cancer, the chatbot offers age-appropriate explanations and emotional support, addressing the limitations of existing solutions. We also conducted a preliminary evaluation of the chatbot’s performance in comparison to two existing solutions: ChatGPT-4 and Microsoft Copilot. The aim was to assess how well each chatbot handled the same prompt in terms of the five standard criteria for evaluating chatbots developed by Liang and Li [16].

## Chatbot Development

### Technology

We chose Dialogflow as the development platform due to its integration with Google Cloud, scalability, and user-friendly interface. We selected Dialogflow ES for its simplicity and lower learning curve, though it has some limitations compared to Dialogflow CX. If scalability requires, migration tools are available. We adjusted the ML Classification Threshold from 0.3 to 0.5 to prevent irrelevant responses from user input. The chatbot was trained using extensive research on childhood cancer and communication best practices.

### Intents

**Fig. 1:**
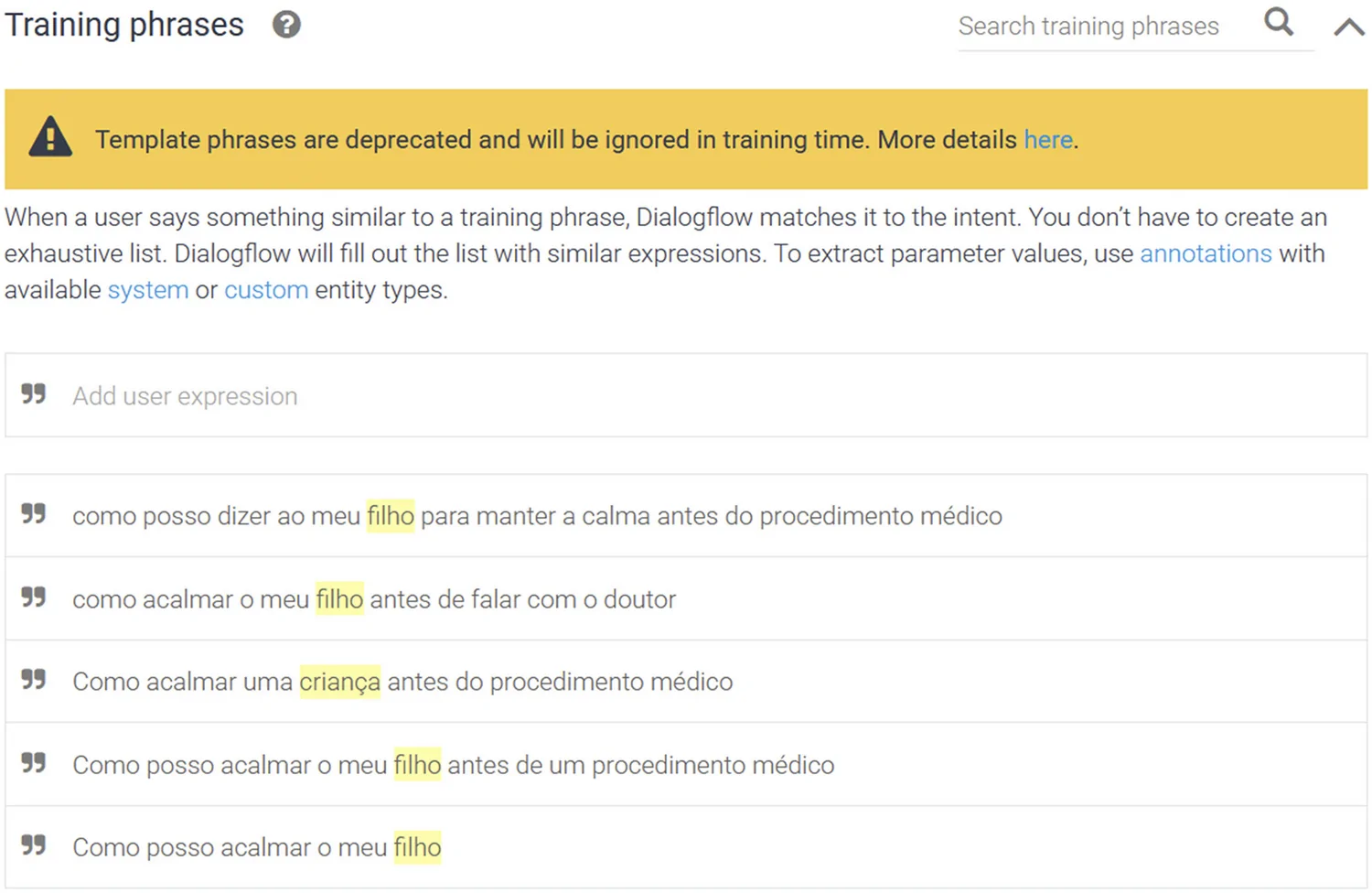
Training phrases for intents in Dialogflow (in Portuguese)

The development of conversational agents, like those on the Dialogflow platform, involves defining clear intents that categorize user intentions and link them to specific actions. Our intents correspond to the main topics addressed in the literature searched, for example, “calm the child,” “emotions,” “explain with age,” “explain cancer,” “talk to husband,” “siblings,” “medications,” “chemotherapy,” and “medical recommendations.” Fig. 1 shows the Dialogflow interface where the training sentences for a specific intent are configured.

### Entities

**Fig. 2:**
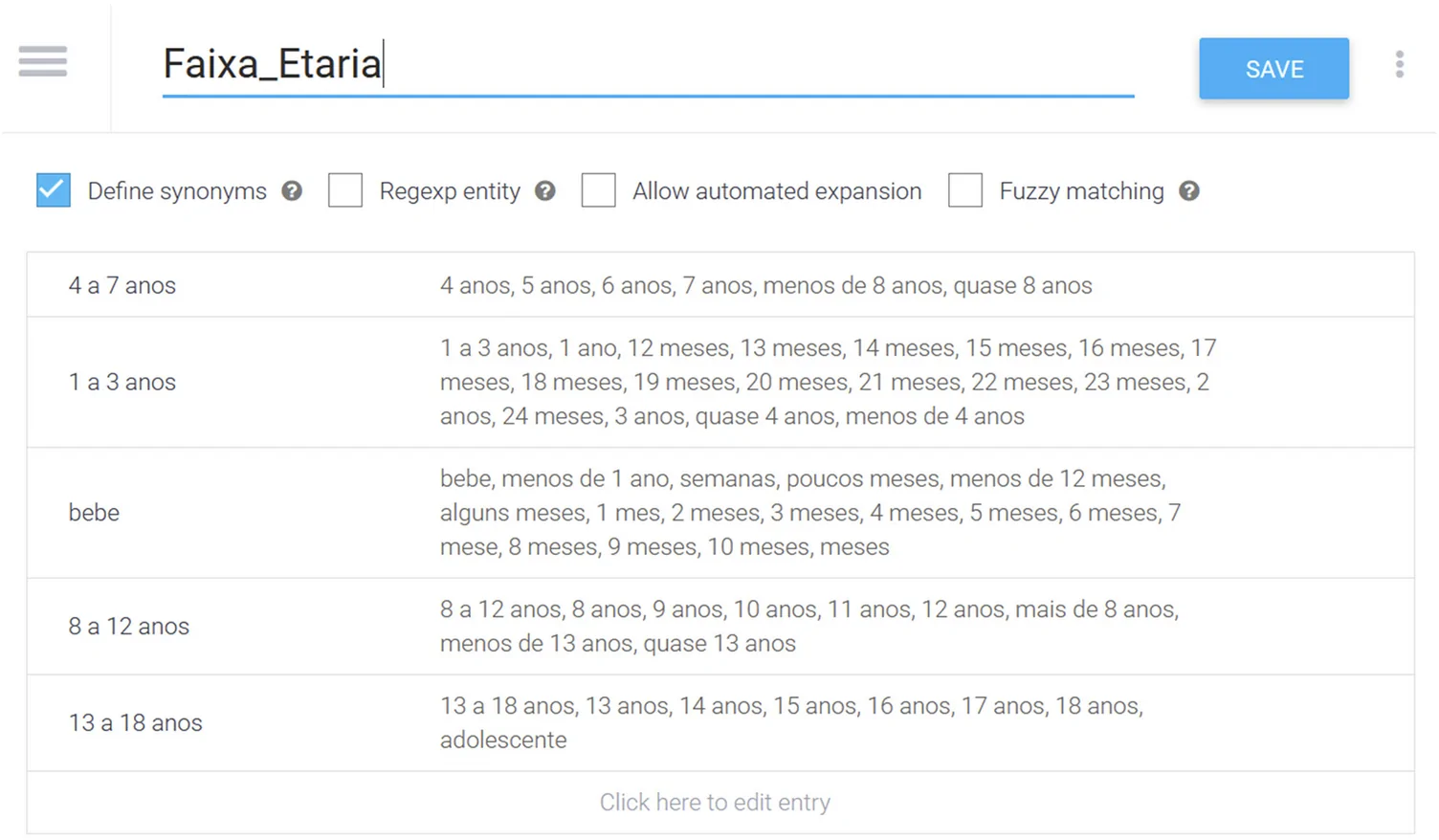
Defining age group entities in Dialoglow (in Portuguese)

In Dialogflow, entities categorize and extract specific information from user inputs, converting it into structured data. Predefined entities recognize common data types, while custom entities allow for tailored classifications. For example, a custom entity was created to classify children and adolescents into age groups, enabling the chatbot to provide age-appropriate responses (Fig. 2). This enhances personalization and relevance. To ensure recognition, entities were tagged in training sentences, allowing the system to process relevant details during real interactions. As a result, the chatbot became a more effective and personalized tool.

### Context

In Dialogflow, contexts help maintain conversational continuity by retaining relevant information from previous interactions. In our chatbot, contexts are used to remember the child’s age, ensuring consistent age-appropriate responses without requiring repetition from parents.

### Fulfillment

**Fig. 3:**
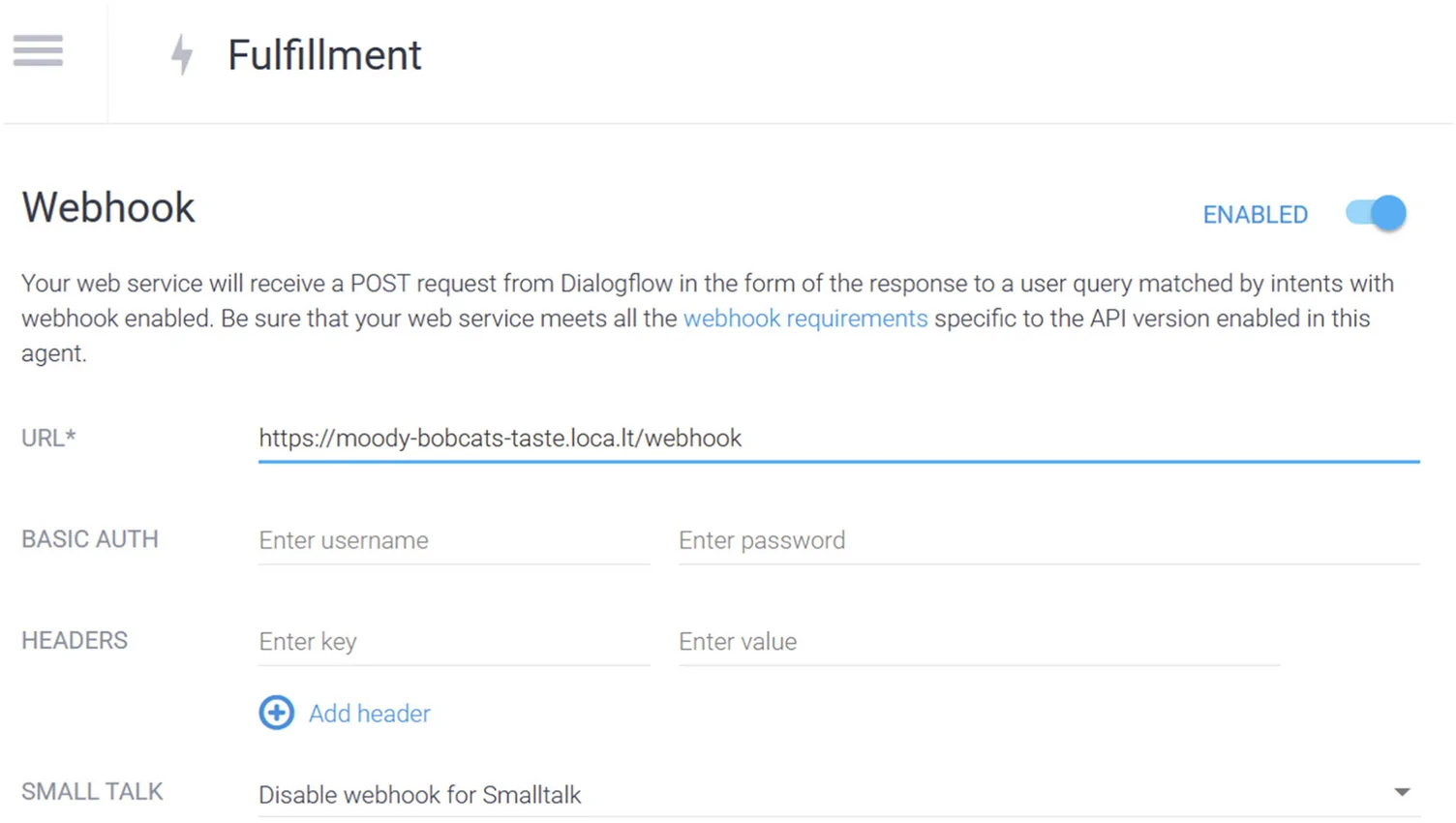
Webhook configuration in Dialogflow

Fulfillment in Dialogflow enables dynamic responses by executing actions or retrieving data beyond static replies. When an intent requires a more complex response, fulfillment sends details (such as intent, action, and parameters) to a webhook. The webhook processes the request and returns a customized response, which Dialogflow delivers to the user. If the webhook fails, Dialogflow provides a predefined fallback response to maintain reliability. Fulfillment was essential for categorizing user queries by age group and providing relevant links, ensuring precise and context-aware answers. Figure 3 illustrates the fulfillment page, which includes a link to the server hosting the webhooks.

### Mobile App

**Fig. 4:**
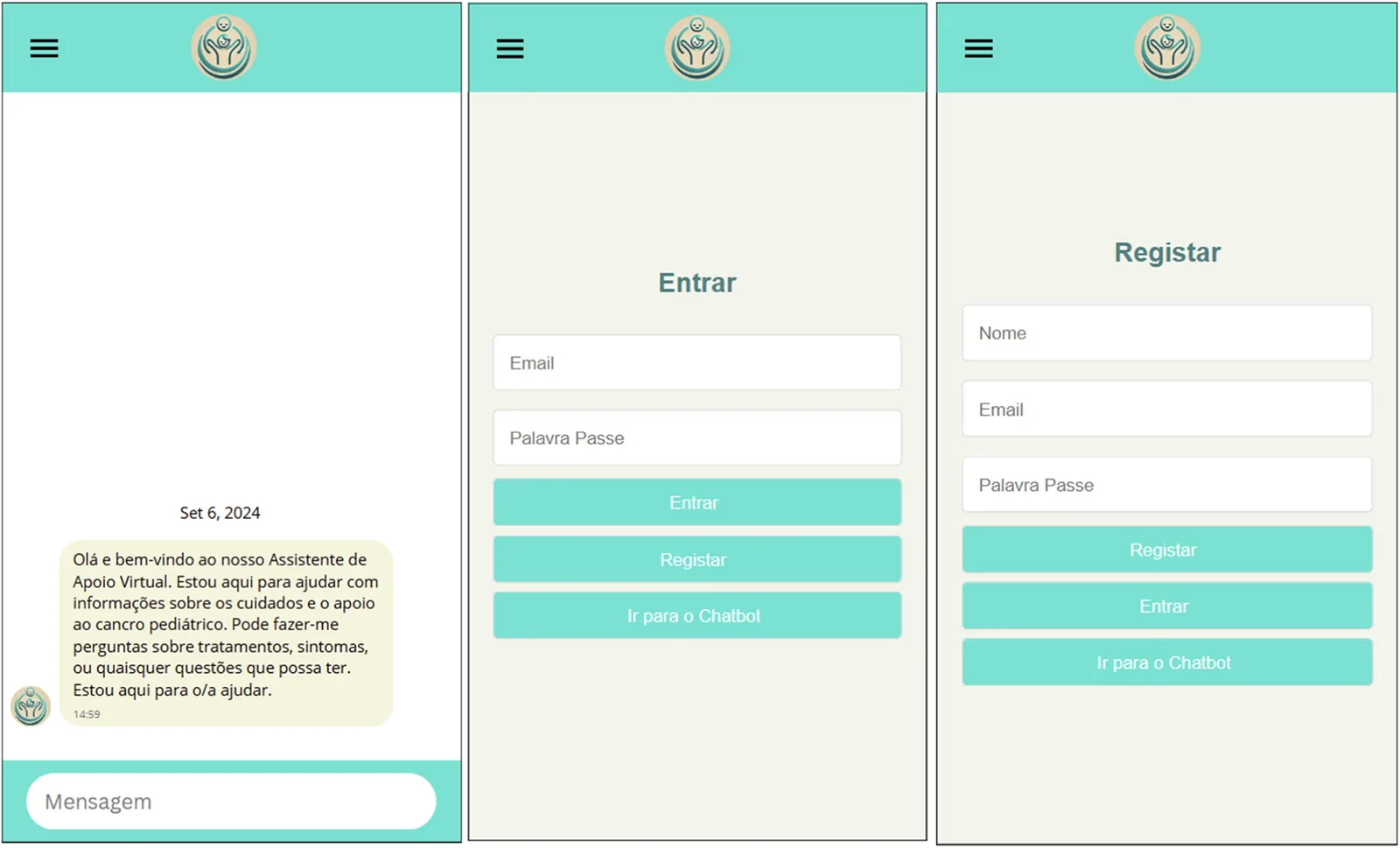
Login and register page mobile app (in Portuguese)

Several technologies were evaluated for developing mobile applications, including Flutter, React Native, Ionic, Xamarin, NativeScript, Apache Cordova, and Framework7. After careful consideration, React Native was chosen due to its cross-platform capabilities, allowing code reuse for both Android and iOS, which significantly reduced development time and costs. React Native provides near-native performance by utilizing native UI components, ensuring a smooth and responsive user experience. Additionally, its active developer community and features like hot reloading enhanced development efficiency, making it the most suitable choice for this project. Figure 4 showcases key mobile app pages, including the chatbot interface, login, and registration screens.

### Website

**Fig. 5:**
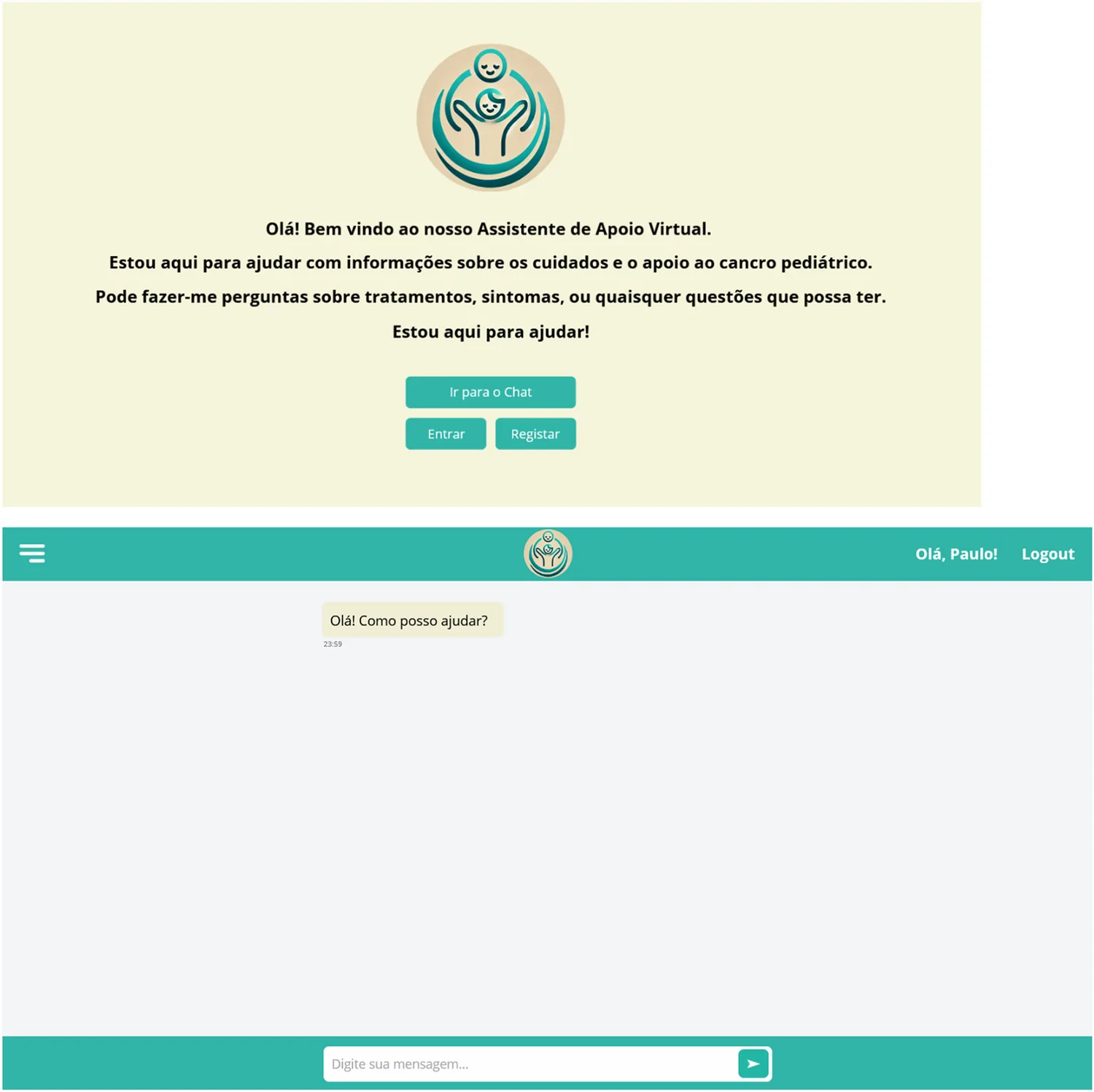
Home page

The website was developed using modern web technologies to provide a seamless and user-friendly experience. The front end was built with Next.js, a React-based framework that supports server-side rendering and static site generation, improving performance and SEO. React’s virtual DOM and component-based architecture manage dynamic interfaces, while Tailwind CSS offers a utility-first approach for responsive and customizable styling. For the mobile version, React Native was used, ensuring compatibility with the React ecosystem. On the backend, Node.js, powered by Google’s V8 engine, ensures scalability and high performance, with Express.js simplifying the creation of RESTful APIs. MongoDB, a NoSQL database, handles large volumes of unstructured data, and Mongoose simplifies database interactions. Security is ensured with bcrypt.js for password hashing, and JWT is used for user authentication and API security. These technologies combine to deliver a high-performance, scalable, and secure web solution. Figure 5 displays key website pages, including the homepage, chatbot interface (guest and authenticated views), and login and registration screens.

### Features

**Fig. 6:**
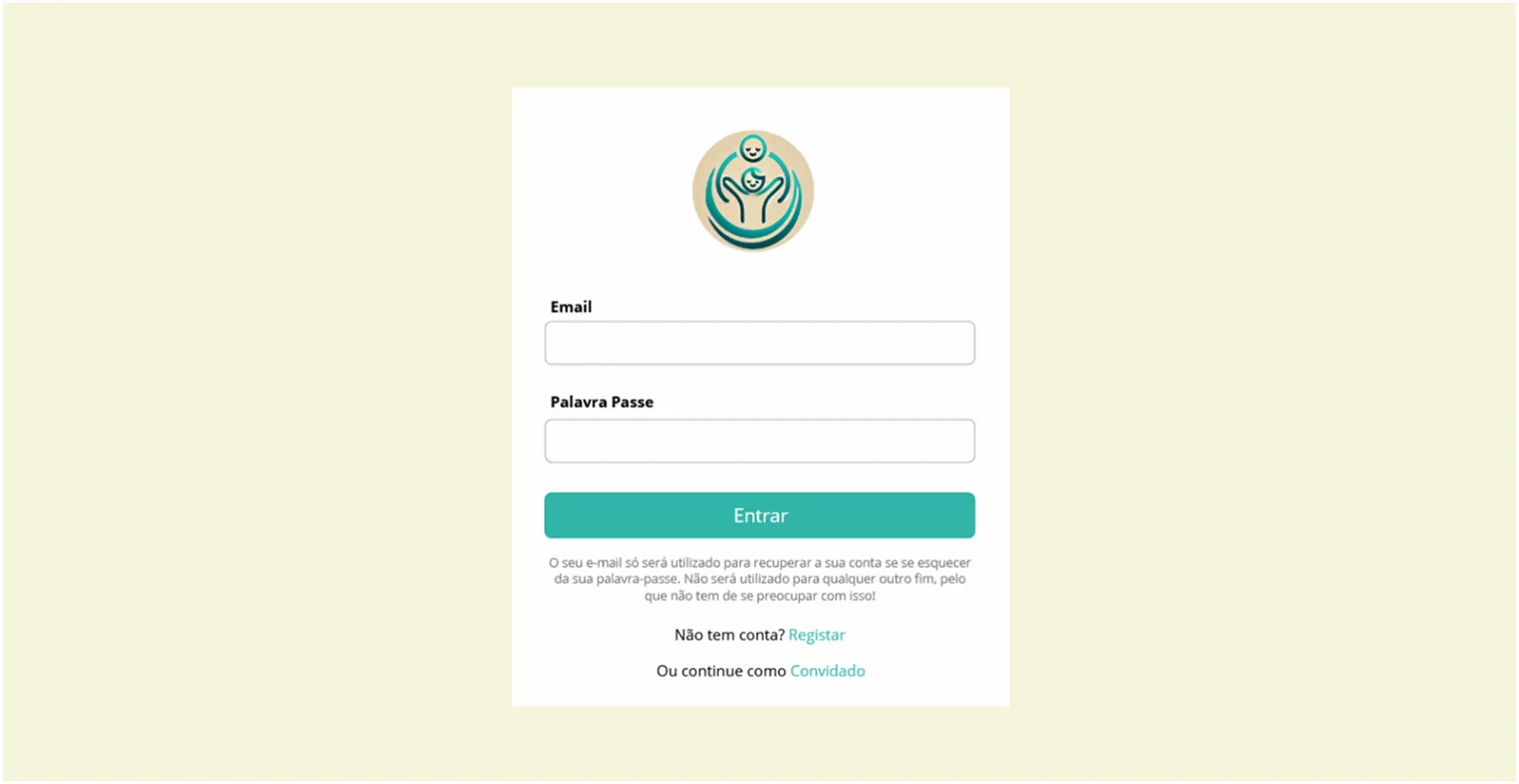
Login page

**Fig. 7:**
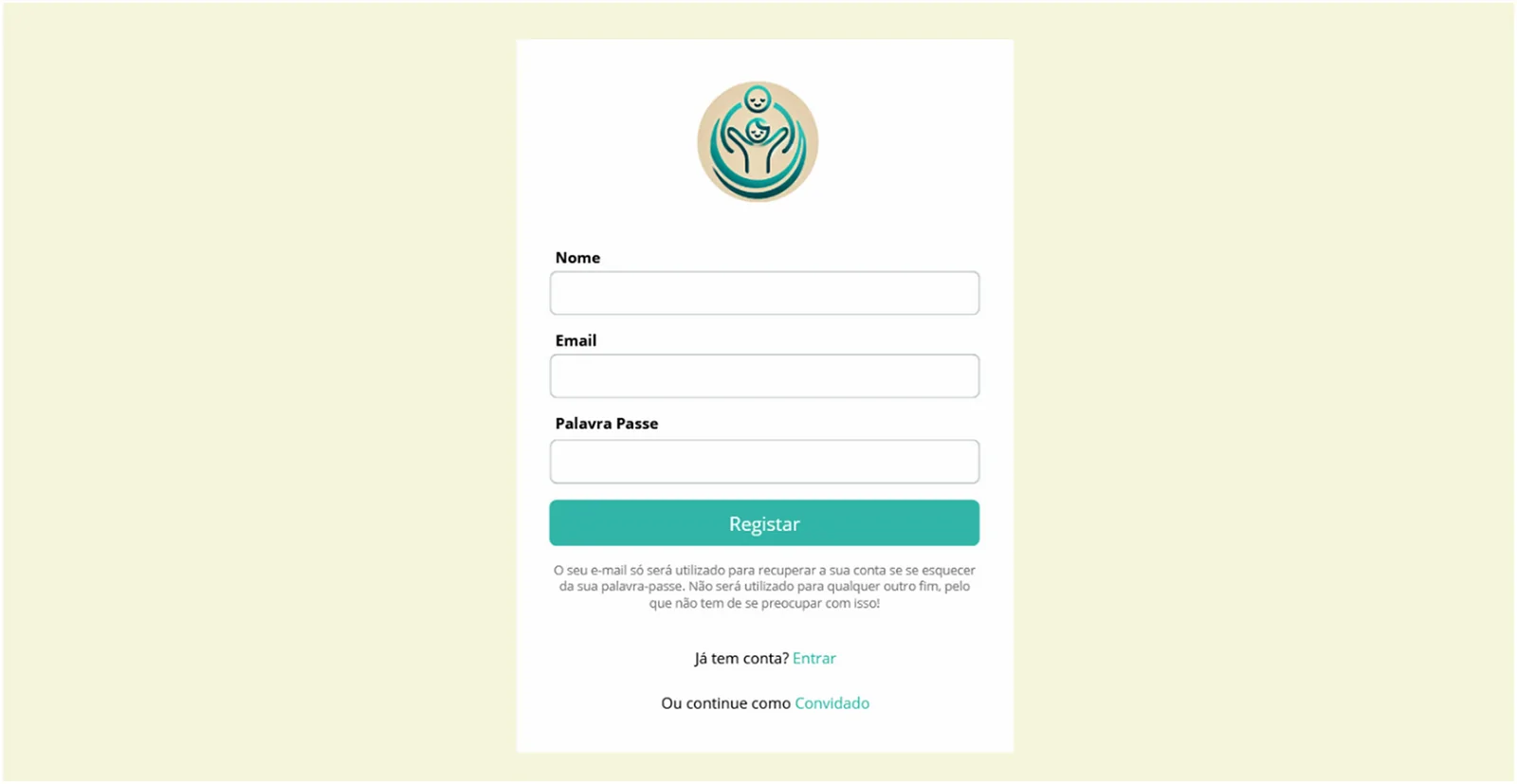
Register page

**Fig. 8:**
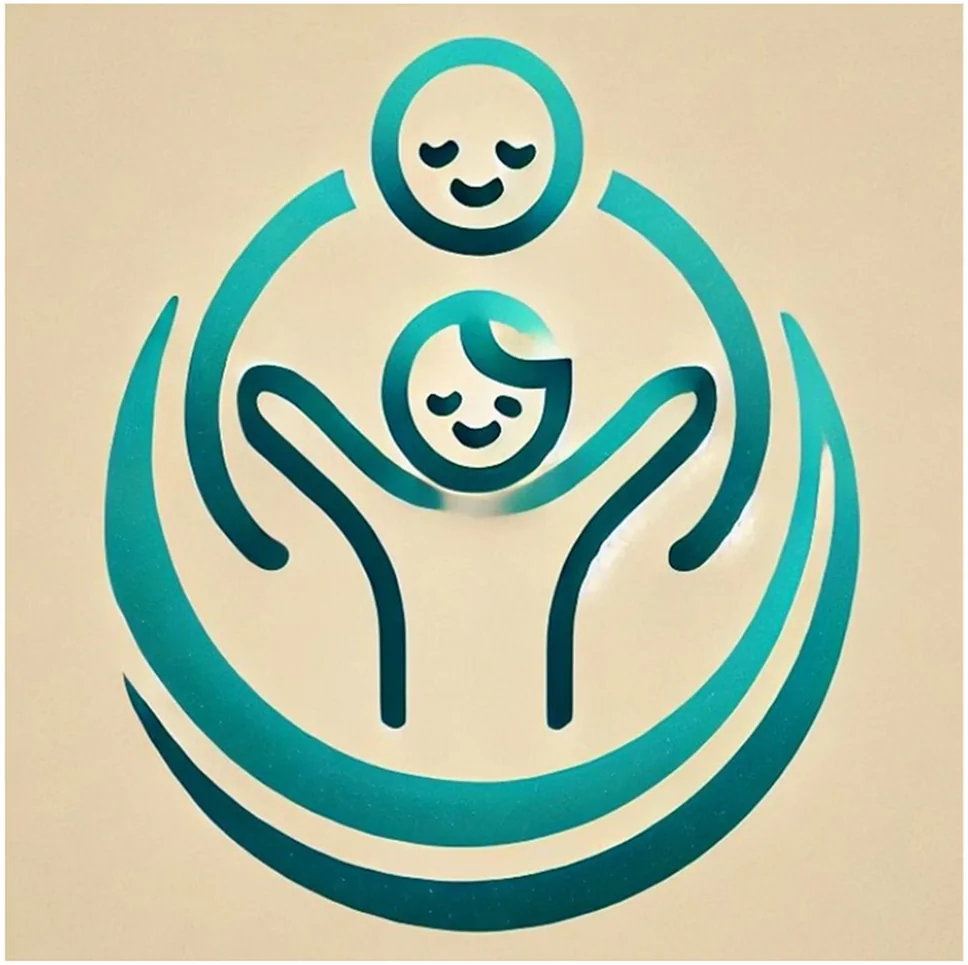
Chatbot Logo

The chatbot was designed to help parents communicate the sensitive topic of childhood cancer to their children in a clear and empathetic way (Fig. 6). It supports both registered users and visitors. During registration, users provide basic details like name, email, and password, with email used only for account recovery to ensure privacy and secure access, as shown on the login and registration pages (Figs. 7 and 8). The chatbot responds in European Portuguese, ensuring culturally relevant communication, and provides helpful links and resources based on user queries. However, it cannot learn autonomously or browse the web, and its responses are limited to pre-trained information. To train the chatbot, we gathered information from various sources specializing in childhood cancer, which provided guidelines on how to talk to children about cancer [17–21]. Figure 8 displays the chatbot logo, featuring abstract figures of a parent and child, symbolizing warmth, trust, and protection, with teal color reinforcing care and support.

## Methods

### Comparative Analysis with Other Chatbots

To assess and compare the quality of chatbot responses, two open-ended questions commonly asked by parents, regarding how to explain the disease to a child and the likelihood of survival, were submitted to three chatbots (our own system and two widely used models) in August 2024. The chatbots were tested under identical conditions and using the same input prompts. All interactions were conducted in Portuguese, with the responses transcribed verbatim and then translated for analysis. Responses were collected in real time via each platform’s standard public interface, with no additional prompts or system modifications applied. The research team evaluated the outputs using five established criteria for the human assessment of chatbot responses [16]: readability (clarity and ease of understanding for general users), relevance (alignment with the question and context), consistency (coherence with existing information), informativeness (depth and accuracy of content), and naturalness (degree to which the response resembles human communication).

## Results

### Question 1–: “How Do I Explain Cancer to a Child?”

#### Response from ChatGPT (OpenAI, Accessed August, 2024) (Unedited Output, Only Translated)

“Explaining cancer to a child can be challenging, but it’s important to be honest, use age-appropriate language and offer comfort and support. Here are some tips on how to approach the subject:

Use simple, clear language: Avoid complex medical terms. You can explain cancer as a kind of ‘fault’ in the body’s cells, which start to grow differently and don’t stop as they should.

Make clear comparisons: Saying that these abnormal cells are like ‘weeds in a garden’ can help the child visualize how they can prevent the body from working well. Explainthat the treatment may make the person feel tired or ill, but it is a way of helping the body get rid of the ‘weeds’.

Offer emotional reassurance: It’s important to reassure the person that cancer is no one’s fault, that it’s not contagious, and that the doctors and family are doing their best to care for the person.

Encourage questions: Allow the child to ask questions and express feelings. This can help them understand the situation better and feel safer.

Maintain routines: Maintaining the child’s daily routine as much as possible can help provide a sense of normalcy and security.

Use resources: Children’s books and videos on the subject can be useful. They are designed to explain cancer in a way that children can understand and process. When explaining cancer, the most important thing is to make the child feel supported and know that they can count on the adults around them for help and comfort.”

#### Response from Microsoft Copilot (Accessed August, 2024) (Unedited Output, Only Translated)

“Explaining cancer to a child can be a challenge, but it’s important to be honest and use simple, age-appropriate language. Here are some tips to help:

Use simple language: Explain that the body is made up of lots of little parts called cells. Sometimes some of these cells don’t work properly and start to grow out of control, forming what we call cancer.

Be honest but optimistic: It’s important to be honest about the disease and the treatment, but also to give hope. Tell them that doctors are doing everything they can to help them get better.

Prepare for change: Explain that the person with cancer may need many treatments, such as chemotherapy or radiotherapy, which can cause side effects such as tiredness or hair loss.

Encourage questions: Let the child ask questions and answer them as clearly as possible. This will help reduce fear and anxiety.

Use visual aids: Children’s books or videos can be useful to explain the condition in a way that the child understands better.

Remember that each child is unique and may react differently. Be available to talk and offer support whenever necessary. If you need more help, consider talking to a health professional or a psychologist who specializes in children.”

#### Response from Our Chatbot (Unedited Output, Only Translated)

“When explaining cancer to a child, it’s important to take their age and development into account. To help me, please tell me your child’s age!

Authors’ data—12 years old.

Children between the ages of 7 and 12 have a better understanding of cancer and how it affects the body. They also have a better understanding of what cells are and how treatments work. It’s easy to talk to your child about cancer as a disease in which some cells in the body grow out of control. The good news is that doctors have specific treatments to control or eliminate these cells. When talking to a child in this age group, don’t be afraid to mention the name ‘cancer’ and try not to leave out any information—children at this age can imagine things that aren’t real and even have thoughts that are worse than reality. It’s also normal for children at this age to be afraid of pain and physical changes, such as hair loss. It’s very important to be honest with your child about what might hurt and explain that these effects are only temporary. Children in this age group are likely to get information about cancer from other sources, such as school or the internet. It’s good for them to share what they’ve heard so they can talk about it and make sure they have all the facts. It’s great that children between the ages of 7 and 12 can prepare for medical procedures and routine changes in advance! This gives them time to adjust and ask questions. It also helps them cope with the idea that the treatment may affect their daily activities, such as going to school. It’s very important to keep the conversation open and let them share their concerns. It’s very important to show children of all ages lots of love and reassurance, especially when they’re going through something as difficult as this. Let them know that even though things are changing, their place in your heart remains the same. It’s very important to create a safe space for conversation and to let children know that it’s OK to feel what they’re feeling. When we do this, we can help them to feel less anxious and more relaxed.”

#### Comparative Analysis of ChatGPT, Microsoft Copilot, and Our Chatbot

1. **Readability**: While ChatGPT and Microsoft Copilot prioritize clarity by using concise language, bullet points, and direct phrasing to make information easier to read quickly, our chatbot uses a more elaborate narrative style. Although this may reduce reading speed, it can foster a deeper emotional connection and encourage user engagement. The extended format enables a more conversational tone and allows complex or distressing information to be introduced in a supportive way.
2. **Relevance**: All three responses are relevant to the question. ChatGPT addresses both the emotional and practical aspects of the condition, offering guidance on maintaining routines and providing emotional support. Microsoft Copilot also provides contextually appropriate advice, particularly about emotional honesty and preparing for side effects. However, Our Chatbot focuses on the developmental stage of the child by asking for their age and tailoring the explanation accordingly.
3. **Consistency**: All three chatbots demonstrate a consistent approach that aligns with the general guidelines for communicating serious topics to children. ChatGPT maintains a consistent tone and message, emphasizing honesty, emotional reassurance, and clarity. Microsoft Copilot also provides straightforward, supportive advice. Similarly, our chatbot closely follows developmental psychology principles, adapting its tone and content to match the cognitive and emotional capacity of the child’s age group.
4. **Informativeness**: Thanks to its age-specific detail, our chatbot provides the most informative responses. It goes beyond general advice by anticipating children’s fears and misunderstandings and identifying potential sources of information, such as school or the internet. It then provides nuanced suggestions on how to handle these issues. Although ChatGPT is informative and uses practical suggestions to guide caregivers, it does not adapt the content for a specific age group. Microsoft Copilot provides the least detail; its advice is more general.
5. **Naturalness**: ChatGPT’s responses are human-like, being both conversational and supportive. By contrast, Microsoft Copilot is clear but adopts a more neutral, clinical tone that is less emotionally engaging. Our chatbot uses emotionally sensitive language to strike a highly natural tone. It mirrors the way a caring adult might speak in real life, thereby enhancing the user’s emotional connection to the advice provided.

### Question 2: “My Son Has Stage 4 Osteosarcoma. How Likely Is He to Survive?”

#### Response from ChatGPT (OpenAI, Accessed August, 2024) (Unedited Output, Only Translated)

“I’m sorry to hear that you’re facing this difficult situation with your son. Osteosarcoma is a type of bone cancer that can be particularly aggressive. When we talk about ‘stage 4’, it usually means thatcancerhas spread to other parts of the body, which can complicate treatment. Survival rates for osteosarcoma vary depending on several factors, including the extent of the disease, the response to treatment, the patient’s general health, among others. In general, stage 4 osteosarcoma presents significant challenges, as metastatic disease is more difficult to treat effectively. Historically, statistics indicate that the 5-year survival rate for patients with metastatic osteosarcoma is around 15% to 30%. However, it is important to emphasize that each case isunique,and these statistics may not apply directly to your child’s case. Advances in treatment, such as targeted therapies and new chemotherapy protocols, have helped to improve the outlook in some cases. It is essential that you talk to the medical team that is looking after your child to get a more accurate assessment based on the specific characteristics of their illness and to discuss the treatment options available. They will be able to provide you with more detailed and personalized informationaboutyour child’s situation. If you have any further questions or need more information, I’m here to help.

Explaining cancer to a child can be challenging, but it’s important to be honest and use simple, age-appropriate language.

Here are some tips that can help:

Use simple language: Explain that the body is made up of lots of little parts called cells. Sometimes, some of these cells don’t work properly and start to grow in an uncontrolled, forming what we call cancer1.

Be honest but optimistic: It’s important to be honest about the disease and treatment, but also to convey hope. convey hope. Tell them that the doctors are doing everything they can to help the sick person get better.

Prepare for changes: Explain that a person with cancer may need many treatments, such as chemotherapy or radiotherapy, which can cause side effects such as tiredness or hair loss.

Encourage questions: Let the child ask questions and answer them as clearly as possible. This helps to reduce fear and anxiety.

Use visual aids: Children’s books or videos can be useful for explaining the disease in a way that the child understands better.

Remember that each child is unique and can react differently. Be available to talk and offer support whenever necessary. If you need more help, consider talking to a health professional or psychologist specializing in children.”

#### Response from Microsoft Copilot (Accessed August, 2024) (Unedited Output, Only Translated)

“I’m sorry to hear about your son’s diagnosis. Stage 4 osteosarcoma, also known as metastatic osteosarcoma, means thatcancer has spread to other parts of the body, such as the lungs or other organs1. The 5-year survival rate for metastatic osteosarcoma is around 27%. This means that about 27 out of every 100 people diagnosed with metastatic osteosarcoma will be alive five years after diagnosis. However, it’s important to remember that these statistics are based on historical data and each case is unique. Factors such as response to treatment, the patient’s general health and advances in treatment can all affect prognosis. Talk to your child’s doctor for a more accurate andpersonalizedassessment. They will be able to provide detailed information about your child’s specific case and discuss the treatment options available. If you need more support or information, I’m here to help.”

#### Response from Our Chatbot (Unedited Output, Only Translated)

“I know this is a very difficult time for you and it’s natural to look for answers in numbers. But every case isunique,and you need to trust that your son has a wonderful, dedicated team who are doing everything they can to give him the best possible treatment and care. Right now, you need to focus on making sure your child feels comfortable and supported. If you have any questions about the treatment or how you can help on a day-to-day basis, don’t hesitate toask!”.

1. **Readability**: ChatGPT provides well-structured, easy-to-read responses that use clear language and simple explanations of medical terminology. Microsoft Copilot also generates concise and direct sentences. In contrast, our Chatbot adopts a more conversational and emotionally attuned tone. Its narrative format provides a more natural reading experience and offers emotional support.
2. **Relevance**: Both initial responses aim to address the user’s primary concern, which is the likelihood of survival in stage 4 osteosarcoma. Both ChatGPT and Copilot provide direct answers to this question, offering general survival statistics and recommending that users consult healthcare professionals for personalized information. By contrast, our chatbot offers a response that validates the parent’s feelings and encourages them to focus on what they can control in the present moment, such as providing care and support, being present, and trusting the medical team.
3. ** Consistency**: Both ChatGPT and Copilot are consistent with current medical literature, presenting survival statistics and emphasizing the importance of personalized medical advice. While this aligns with clinical sources, communicating prognosis data directly, particularly in the absence of professional emotional support, may not be the most appropriate approach when speaking to parents who are facing a highly distressing diagnosis. In contrast, Our Chatbot provides a psychosocially informed response that prioritizes emotional support.
4. **Informativeness**: Of the three, ChatGPT is the most informative. It discusses survival rates, explains the implications of stage 4, and highlights the role of individual variation. It also mentions emerging treatments. Microsoft Copilot also provides information, albeit in a more concise and neutral manner. While both models deliver concrete data, they may assume a level of emotional readiness that not all users possess. In contrast, our Chatbot focuses on psychosocial support, emphasizing an equally important aspect of informativeness grounded in users’ emotional needs rather than clinical statistics.
5. **Naturalness**: ChatGPT combines emotional sensitivity with medical explanations. By contrast, Copilot is more neutral and distant. Our chatbot stands out thanks to its natural, human-like manner of speaking and its acknowledgement of how difficult the situation is. This kind of tone can make parents feel more supported.

## Discussion and Conclusion

### Discussion

This project led to the development of an innovative chatbot designed to assist parents in communicating complex medical information to Portuguese children with cancer. In 2023, a Portuguese physician highlighted the potential of ChatGPT to provide age-appropriate answers, enhancing parent–child communication in childhood cancer [22]. In response to this need, our chatbot is tailored to the developmental stages of young patients. It asks the child’s age and then delivers clear, precise, and reliable information that is relevant to their age. It also offers additional resources for further research, improving the accessibility and applicability of the shared information. A key feature of this chatbot is its focus on childhood cancer, ensuring that the responses are specifically tailored to the needs of families affected by this condition. The chatbot avoids presenting statistics or potentially distressing data, instead prioritizing positive, constructive, and age-appropriate messages about treatment and care.

This chatbot represents a pioneering initiative in childhood cancer, offering a more tailored and relevant approach compared to existing systems. The only comparable study we found aimed at improving parent–child communication in childhood cancer is the ARCH chatbot [23]. ARCH consists of two components: a Child Bot, which helps children express their concerns, and an Expert Bot, which guides parents based on the child’s inputs. This chatbot was co-designed with children and parents, and its development was validated by 15 pediatric care experts who identified key challenges and helped refine the prototype. These experts emphasized three main roles for ARCH––Agents for Reinforcing Child–parent Health communication––providing an expressive outlet for children, offering reassurance and child-specific guidance to parents, and serving as an assessment tool for healthcare providers to evaluate the communication practices of parent–child dyads. In the case of our chatbot, we conducted a comparative analysis based on standard criteria for the human evaluation of chatbots, comparing it with existing solutions. According to our analysis, our chatbot outperforms traditional models by addressing the unique needs of childhood oncology patients.

### Conclusion

This chatbot demonstrates the potential of advanced technology to support families and enhance communication in complex medical situations, showcasing the transformative impact technology can have on modern medicine. There are numerous opportunities for further research and development of this chatbot. Conducting a comparative analysis with healthcare professionals experienced in the target domain and, in the case of positive feedback, carrying out real-world user trials will be essential to assess the chatbot’s effectiveness and gather feedback to improve the emotional and informational support it provides to families affected by childhood cancer. Plans include integrating features such as saving conversation history and enabling users to store important messages for future reference. Expanding the content of the chatbot to cover additional topics such as daily life, new treatments, and emotional wellbeing would further enhance its usefulness and make it an even more comprehensive resource for families dealing with childhood cancer treatment. These planned enhancements will not only improve the chatbot’s functionality but also solidify its role as an important support tool for childhood cancer families.

### Practice Implications

The development and future implementation of this chatbot can help improve childhood cancer care for both healthcare professionals and families. For healthcare professionals: (1) It can improve communication in medical settings by acting as a bridge between healthcare providers and families, ensuring that medical information is communicated in an understandable way. (2) Although designed for childhood cancer, the chatbot framework has the potential to be adapted for other medical conditions, extending the reach of AI-driven support in healthcare contexts. For families: (1) this tool provides parents with clear, empathetic, and age-appropriate responses to help them prepare their conversations with their children. (2) Its accessibility through a mobile app and website allows parents to seek guidance anytime, anywhere, providing ongoing support without the need for immediate professional intervention and providing answers to their child’s doubts and concerns.

## Data Availability

Data are available upon request.
